# Biomechanical Assessment Methods Used in Chronic Stroke: A Scoping Review of Non-Linear Approaches

**DOI:** 10.3390/s24072338

**Published:** 2024-04-06

**Authors:** Marta Freitas, Francisco Pinho, Liliana Pinho, Sandra Silva, Vânia Figueira, João Paulo Vilas-Boas, Augusta Silva

**Affiliations:** 1 Escola Superior de Saúde do Vale do Ave, Cooperativa de Ensino Superior Politécnico e Universitário, Rua José António Vidal, 81, 4760-409 Vila Nova de Famalicão, Portugal; francisco.pinho@ipsn.cespu.pt (F.P.); liliana.pinho@ipsn.cespu.pt (L.P.); sandra.silva@ipsn.cespu.pt (S.S.); vania.figueira@ipsn.cespu.pt (V.F.); 2 H^2^M—Health and Human Movement Unit, Polytechnic University of Health, Cooperativa de Ensino Superior Politécnico e Universitário, CRL, 4760-409 Vila Nova de Famalicão, Portugal; 3 Center for Rehabilitation Research (CIR), R. Dr. António Bernardino de Almeida 400, 4200-072 Porto, Portugal; augusta.silva@ess.ipp.pt; 4 Porto Biomechanics Laboratory (LABIOMEP), 4200-450 Porto, Portugal; 5 Department of Medical Sciences, University of Aveiro, 3810-193 Aveiro, Portugal; 6 School of Health Sciences, University of Aveiro, 3810-193 Aveiro, Portugal; jpvb@fade.up.pt; 7 Centre for Research, Training, Innovation and Intervention in Sport (CIFI2D), Faculty of Sport, University of Porto, 4200-450 Porto, Portugal; 8 Department of Physiotherapy, School of Health, Polytechnic of Porto, 4200-072 Porto, Portugal

**Keywords:** chronic stroke, assessment, non-linear, kinetic, kinematic, EMG

## Abstract

Non-linear and dynamic systems analysis of human movement has recently become increasingly widespread with the intention of better reflecting how complexity affects the adaptability of motor systems, especially after a stroke. The main objective of this scoping review was to summarize the non-linear measures used in the analysis of kinetic, kinematic, and EMG data of human movement after stroke. PRISMA-ScR guidelines were followed, establishing the eligibility criteria, the population, the concept, and the contextual framework. The examined studies were published between 1 January 2013 and 12 April 2023, in English or Portuguese, and were indexed in the databases selected for this research: PubMed^®^, Web of Science^®^, Institute of Electrical and Electronics Engineers^®^, Science Direct^®^ and Google Scholar^®^. In total, 14 of the 763 articles met the inclusion criteria. The non-linear measures identified included entropy (n = 11), fractal analysis (n = 1), the short-term local divergence exponent (n = 1), the maximum Floquet multiplier (n = 1), and the Lyapunov exponent (n = 1). These studies focused on different motor tasks: reaching to grasp (n = 2), reaching to point (n = 1), arm tracking (n = 2), elbow flexion (n = 5), elbow extension (n = 1), wrist and finger extension upward (lifting) (n = 1), knee extension (n = 1), and walking (n = 4). When studying the complexity of human movement in chronic post-stroke adults, entropy measures, particularly sample entropy, were preferred. Kinematic assessment was mainly performed using motion capture systems, with a focus on joint angles of the upper limbs.

## 1. Introduction

The integrated concept of the human movement system (HMS) results from a broad physiological network comprising interactions between the neurological, musculoskeletal, cardiovascular, respiratory, tegumentary, and metabolic systems in order to move the body or parts of it [1]. This interaction fluctuates in a continuous, complex, and non-linear manner [2,3], acting on different time scales in response to different contextual conditions to adapt and maintain harmonious movement based on its characteristic variability [3].

Motor variability is an inherent feature of HMS, enabling a variety of solutions and strategies during one task [4], and providing flexibility to the response depending on context or unexpected environmental changes [2]. Thus, human movement is also endowed with complexity, consisting of the ability to perform a specific task with different strategies over a period and the regularity of the inherent variability pattern [5]. These properties should allow movements without rigid patterns [4] to respond to small perturbations and task demands [6]. Thus, all functional movements ideally have variability and complexity, translating into a state between high variability with multiple movement options and complete repeatability [7]. This needs to be described and studied objectively for better understanding.

Linear measures are limited in explaining the variability of human movement and cannot fully describe its characteristics [8], which are predominantly non-linear [9]. The use of non-linear measures allows the description of small and subtle changes that depend on time and the environment, which, according to a linear approach, could be diluted by the averaging of global data, making them imperceptible, despite their existence [10]. 

Both approaches, linear and non-linear, are complementary and should be considered in the analysis of human movement, as both are sensitive to small perturbations that occur and affect performance and motor capacity [6]. According to a linear perspective, any typical movement without deviations that could be considered as errors represents human movement with optimal variability [2]. This contrasts with the perspective of the non-linear approach, where movement with ideal variability is actually fundamental for individuals to perform a range of variations to adapt to small perturbations induced by the environment [2,11]. A non-linear approach appears to be more consistent with dynamic human systems, as suggested by Montesinos et al. [12], and with the way interventions should be advocated for in this domain [2,7,13]. 

Among neuromotor disorders, stroke is the second leading cause of death and the third leading cause of disability worldwide [14]. It is commonly characterized by impairments in movement complexity and variability, resulting in functional task difficulties and reduced patient autonomy due to limitations in motor and postural control [15,16]. Linear approaches may provide limited information about the motor control system’s response to change and may not include inherent aspects of the complex movement system [4,17]. Recognizing that variability analysis can enhance our understanding of adaptive strategies and the overall behavior of dynamic systems in post-stroke recovery, the investigation of non-linear measurements has emerged as a novel approach for the better interpretation of the neuromotor expression of this condition [4].

To our knowledge, there has not been a comprehensive review of the non-linear methods used to study sensorimotor recovery after stroke, despite emerging studies utilizing non-linear analysis to study stroke populations [18,19,20]. Understanding sensorimotor recovery is crucial for neurorehabilitation, particularly post-stroke recovery. Identifying measures and methods can aid in compiling data, interpreting the mechanisms underlying motor recovery, and guiding more effective intervention strategies [21].

Thus, the main objective of this scoping review was to summarize the non-linear measures used in the analysis of kinetic, kinematic, and electromyographic (EMG) data of human movement after stroke. The secondary aim was to summarize the methodological considerations, namely participant characteristics, studied tasks, measurement instruments and kinetic, kinematic, and EMG variables.

Review questions

The main review question was “What non-linear measures are used in the processing of kinematic, kinetic, and EMG data in the assessment of human movement after chronic stroke?”

The review sub-questions are listed as follows:

1. What tasks were performed in the identified studies?
2. What laboratory instruments were used to collect kinematic, kinetic, and EMG data in the identified studies?
3. Which kinematic, kinetic, and EMG variables were included in the identified studies?

## 2. Materials and Methods

Our scoping review was conducted in accordance with the Preferred Reporting Items for Systematic Reviewers and Meta-Analysis extension for Scoping Reviews (PRISMA-ScR) [22]. The Supplementary Material protocol was registered on the Open Science Framework, in which the review questions and the methodology were specified, https://osf.io/r3pe9 (accessed 28 July 2023). The following supporting information can be downloaded from https://doi.org/10.17605/OSF.IO/R3PE9 (accessed on 7 July 2022).

### 2.1. Eligibility Criteria

Eligibility criteria were defined a priori using the acronym PCC (population, concept, and context) according to the methodology of the Joanna Briggs Institute (JBI) [23] (Table 1). 

Experimental and epidemiological study designs published in English or Portuguese were eligible for inclusion. Systematic, narrative, or scoping reviews (to avoid duplication of data), letters, editorials, and qualitative methodological designs were excluded.

### 2.2. Search Strategy

Relevant studies were identified by searching through the PubMed^®^, Web of Science^®^, Institute of Electrical and Electronics Engineers^®^ (IEEE) and Science Direct^®^ databases. In addition to these databases, the scholarly literature web search engine, Google Scholar^®^, was also used. To identify other eligible studies, the reference lists of the original research articles and reviews on the topic were manually reviewed. Studies published from 1st January 2013 to 12 April 2023 were included.

The search strategy for PubMed^®^ was as follows: stroke AND (measure OR measurement OR evaluation OR analysis OR assessment) AND (non-linear OR nonlinear OR entropy OR Lyapunov OR “nonlinear variables” OR tools OR dynamic OR variability) AND (“human movement” OR motion) NOT (EEG OR cardiac). Two reviewers independently carried out the search.

### 2.3. Selection of Evidence Sources

The selection of evidence sources considered the PCC acronym, purpose, and research questions. Both investigators (MF and LP) performed the search simultaneously in the same databases using the defined strategies. No discrepancies were found during the data extraction process, which was imported into Endnote^®^ where duplicates were removed. To facilitate the screening process and confirm the presence of any duplicates not identified by the software (Endnote version 20.0), all extracted articles were imported into Microsoft Excel. 

Two independent reviewers analyzed the same 25 abstracts that met the eligibility criteria in a pilot test (the first 25 titles/abstracts of the PubMed database) [25]. The screening process did not begin until a consensus of at least 75% was reached [25].

Two reviewers screened the titles and abstracts and categorized studies as either ‘included’ or ‘excluded’. This stage allowed for the identification of articles for full-text screening. Two independent reviewers (MF and LP) extracted the data, and any disagreements were resolved by a third reviewer (SS).

### 2.4. Data Extraction

Two authors (MF and LP) independently extracted the relevant data using a draft charting table adapted from the original JBI template. Data were extracted according to the following categories: authors, year of publication, study design, participant characteristics (n, sex, age, side lesion, stroke type, time post-stroke), tasks studied, assessment instruments, kinetic, kinematic and EMG variables used to obtain the non-linear measures, and non-linear measures. Disagreements were resolved by a third author.

### 2.5. Data Presentation

To summarize the extracted data, a narrative report was produced on the following outcomes: tasks, assessment instruments, kinetic, kinematic and EMG variables, non-linear measures, and non-linear parameters. The results were expressed in relation to the main question of the study, complemented by a tabular form summarizing the main findings.

## 3. Results

**Figure 1:**
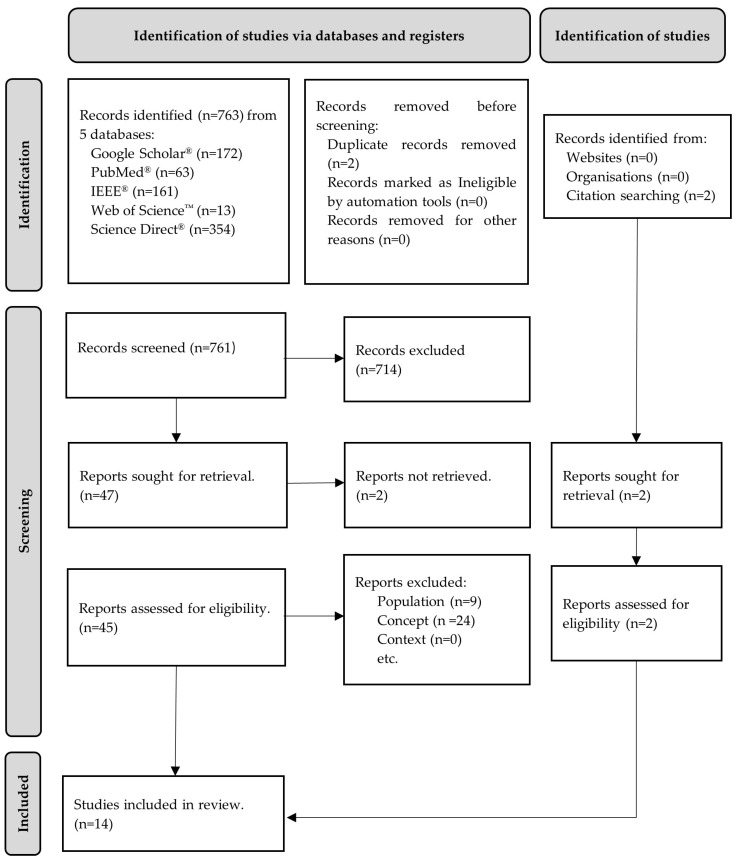
Flow diagram for the scoping review process adapted from the PRISMA-ScR statement [26].

A total of 763 articles were identified—761 records via a database search and 2 additional articles via a manual search of the reference lists. After removing 2 duplicates, 761 records remained. The screening of titles and abstracts resulted in the removal of 714 articles, while 2 articles were not retrieved, leaving 45 for full-text analysis. Of these, 33 were excluded after full-text analysis because they did not meet the inclusion criteria, namely the population (n = 9) and concept (n = 24). The remaining 14 articles were included in this review. The results of the search were presented in a PRISMA-ScR flowchart, as shown in Figure 1.

Table 2 and Table 3 summarize the details of the reviewed articles, including participant characteristics, tasks, assessment instruments, kinetic, kinematic and EMG variables, non-linear measures, and non-linear parameters.

### 3.1. Participant’s Characteristics

Of the 14 included studies, 6 only included post-stroke subjects [19,28,30,33,34,35], and 8 also included healthy subjects [18,27,29,31,32,36,37,38]. The sample size of chronic post-stroke participants ranged from 3 [37] to 23 [29]. 

A total of 150 chronic post-stroke subjects, 102 men and 51 women, with a mean age of 52.33 years, were included in the studies. All the subjects were at a chronic stage, ranging from 12.67 [37] to 177.34 [18] months after stroke, and presented with a single and unilateral [18,19,27,28,30,33,35,37,38] or multiple and unilateral strokes [29]. Two studies did not report lesion characteristics [32,39].

The side of the body most affected was reported in 11 studies [19,27,28,29,30,32,33,34,35,36,37], with a total of 48 right-sided and 71 left-sided strokes. According to the type of stroke, four trials included both ischemic and hemorrhagic stroke [19,30,32,37] and three trials analyzed only ischemic stroke [27,28,33]. Overall, seven trials did not report the type of stroke [18,29,31,34,35,36,38]. Only one study reported the location of the stroke [19,33].

The Mini Mental State Examination (MMSE) was used in two trials to ensure an understanding of the instructions in the required tasks [19,37]. To assess sensorimotor impairment, the Fugl–Meyer Upper Extremity (UE_FM) was used in five studies [27,28,33,36,37], while the Fugl–Meyer Lower Extremity (LE_FM) was used in four studies [18,29,31,35]. Other clinical measures were included in three trials, namely the Motor Activity Log [28], the Rivermead Mobility Index (RMI) [29], and the Stroke Impact Scale (SIS)—Hand Function [19]. The Modified Ashworth Scale (MAS) was used to quantify spasticity in six studies [27,29,32,34,36,37]. Only two studies did not identify a clinical measure to meet inclusion criteria or sample characterization [30,38].

### 3.2. Tasks

**Figure 2:**
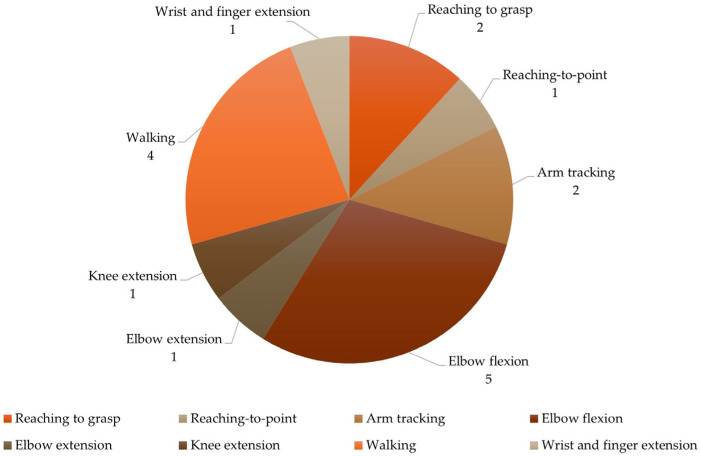
Characterization of the tasks.

Considering the tasks (described in Table 2), nine studies focused on UL and four on LL assessment (Figure 2). Regarding UL tasks, two studies analyzed reaching to grasp [27,28] and one analyzed reaching to point [33]. Arm tracking movement was assessed by two studies [30,32]. Five studies focused on elbow flexion [30,32,34,36,37], one on extension [37], and one on wrist and finger extension upward (lifting) [19]. The remaining studies analyzed knee extension [29] and walking [18,31,35,38].

### 3.3. Assessment Instruments

**Figure 3:**
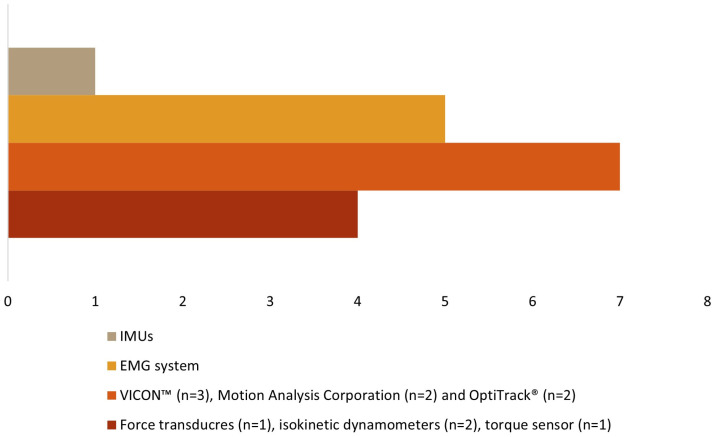
Types of assessment instruments.

Table 3 shows the instruments used to explore kinematic, kinetic, and EMG variables in the 14 studies. Kinetic data were collected in four studies [19,29,30,34], kinematic data were collected in eight studies [18,27,28,31,32,33,35,38], and sEMG data were collected in five studies [30,32,34,36,37] (Figure 3).

Force transducers [19], isokinetic dynamometers [29,30], and a torque sensor [34] were used to evaluate the kinetic data. The muscular activity was assessed using a sEMG system [30,32,34,36,37]. Three different motion capture systems were used for kinematic data: VICON™ [27,28,33], Motion Analysis Corporation [31,35], and OptiTrack^®^ [32,38], with several cameras, ranging from 6 [38] to 12 [27,28,33] in number. One study did not report the number of cameras [32]. The number of reflective markers ranged from 2 [32] to 67 [27,28,33], as different anatomical references were considered within each identified segment according to each author (see Table 3). One study [18] used wireless inertial units (IMUs) in lower limb segments and the pelvis to collect kinematic data.

### 3.4. Kinetic, Kinematic and EMG Variables

**Figure 4:**
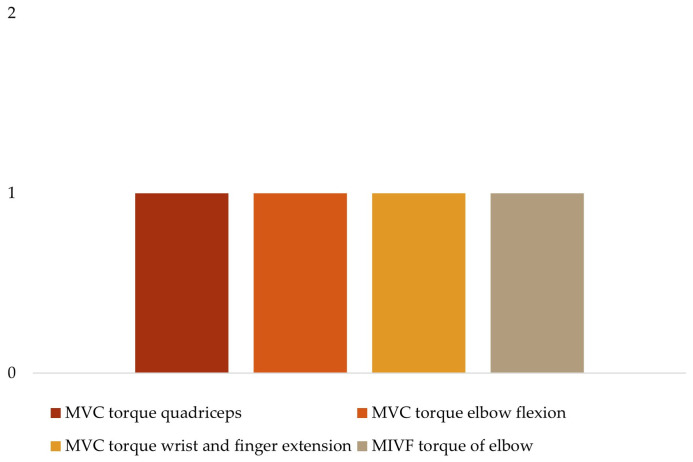
Kinetic variables.

**Figure 5:**
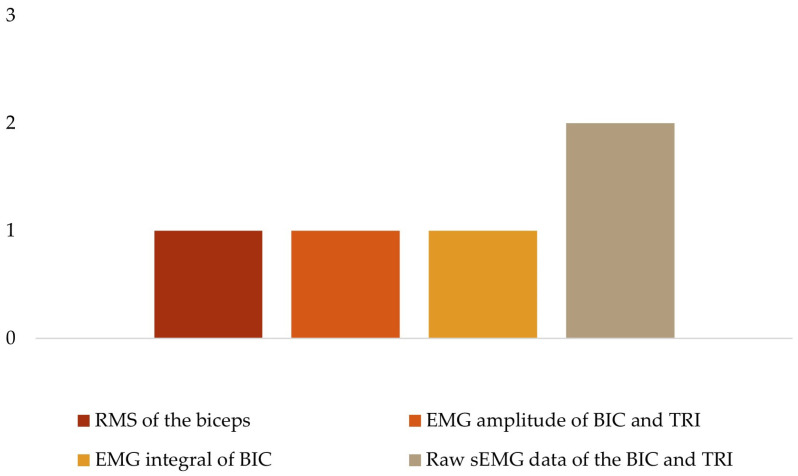
EMG variables.

To analyze the human movement data, different instruments were used to collect several variables (Table 3). For kinetic data (Figure 4), three studies quantified the maximal voluntary contraction (MVC) torque and power of the quadriceps [29], elbow flexion [34], and wrist and finger extension [19]; one study used the maximum isometric voluntary extension (MIVE) torque of the elbow [30]; and another used the maximum isometric voluntary flexion (MIVF) torque of the elbow [30]. Regarding sEMG data (Figure 5), one study each measured muscle activity using the root mean square (RMS) of the biceps (BIC) [34], the EMG amplitude of the BIC and triceps (TRI) [32], the EMG integral of the BIC [36]; the remaining two studies used raw sEMG data of the BIC and TRI [30,37].

**Figure 6:**
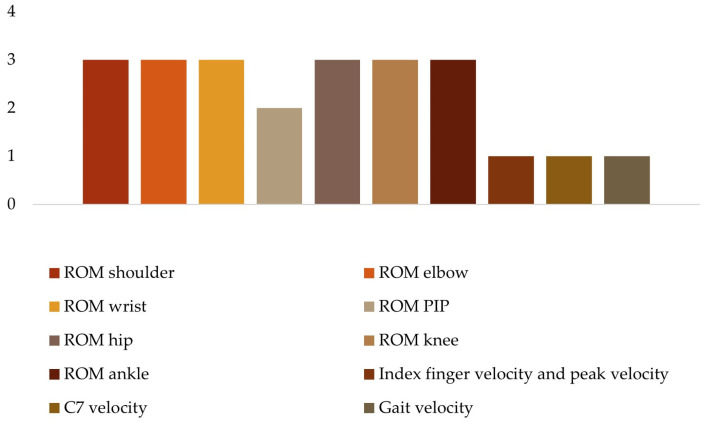
Kinematic variables.

To analyze the kinematic data (Figure 6), four studies considered the range of motion (ROM) of the upper limb, namely the shoulder [27,28,33], elbow [27,28,32,33], wrist [27,28,33] and proximal interphalangeal (PIP) index finger [27,33]. The range of motion of the lower limb was considered in three studies, specifically the hip [18,35], knee [18,35,38], and ankle [18,35,38]. Only three studies included spatial–temporal kinematic parameters such as index finger velocity and peak velocity [33], C7 velocity [31], and gait velocity [35].

### 3.5. Non-Linear Measures

**Figure 7:**
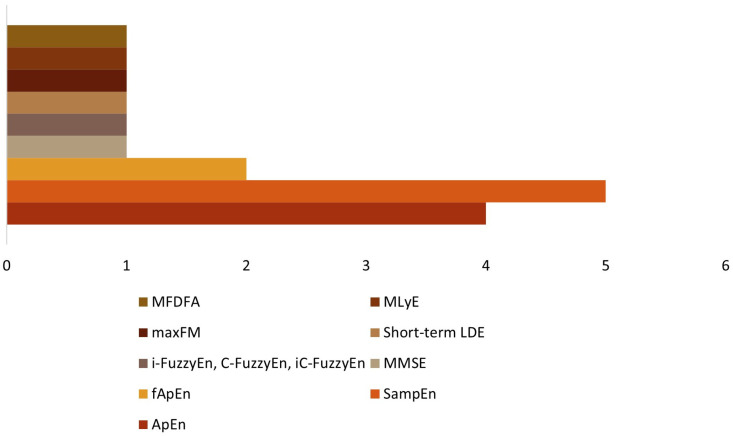
Non-linear measures.

Considering the units included in this review, 11 studies used entropy measures (Table 3). Approximate entropy (ApEn) was used in four studies [27,28,32,33]; sample entropy (SampEn) was in five studies [19,29,32,34,36]; fuzzy approximate entropy (fApEn) was used in two studies [30,32], and multivariate multiscale entropy (mMSE) was used in one study [18]. One study [37] considered the instantaneous fuzzy entropy (i-FuzzyEn), the cross-fuzzy entropy (C-FuzzyEn), and the instantaneous cross-fuzzy entropy (iC-FuzzyEn). The three remaining studies utilized the short-term local divergence exponent (LDE) and the maximum Floquet multiplier (maxFM) [31], the maximum Lyapunov exponent (MLyE) [35], and the multifractal detrended fluctuation analysis (MFDFA) [38]. Figure 7 summarizes the non-linear measures utilized in the studies included.

### 3.6. Non-Linear Measures Parameters

For each of the non-linear measures, the included authors generally described their parameters in detail. Specifically, seven studies mentioned the dataset length (*N*) [18,19,29,30,31,34,36], eleven studies described the specific pattern length (*m*) [18,19,27,28,29,30,32,33,34,36,37], and ten studies specified the criterion of similarity (*r*) [18,19,27,28,29,30,33,34,36,37]. Time series were reduced to *N* points by analyzing between 20 [38] and 5000 points [32]. The time delay between related series of observations (lag) was described in three studies [27,28,33]. Furthermore, there is variation in the length of *m*, ranging from 2 (n = 10) [18,19,27,28,30,32,33,34,36,37] to 3 (n = 1) [29], and *r* ranging from 0.2 [18,19,27,28,29,30,33,37] to 0.25 [34,36]. In the studies that included lag (n = 3), the value remained constant at 1 [27,28,33]. Additionally, the studies had varying numbers of trials and lengths, ranging from 2 [29] to 18 trials/condition [32], and from 3 s [36] to 3 min [38], respectively. Only nine studies described the number of attempts/times analyzed in non-linear analyses [18,19,27,28,29,31,33,34,36].

## 4. Discussion

This scoping review aimed to summarize information on the current published research related to the non-linear measures used in the kinetic, kinematic, and EMG data analysis of human movement after stroke. 

Considering that everyday functional tasks involve the continuous adaptation of movement patterns to meet their dynamic demands, the study of variability in everyday tasks is a key point in human movement analysis due to its non-linear behavior. This information can provide a greater opportunity to better understand the mechanisms underlying an impaired sensorimotor system and may suggest better interventions to improve movement adaptability while encouraging reflection on key findings to be considered in future research. Therefore, this discussion is divided into different aspects: (1) sample characterization; (2) tasks, non-linear measures, and non-linear parameters; (3) assessment instruments; and (4) kinematic, kinetic, and EMG variables.

### 4.1. Sample Characterization

In the included studies, the characteristics of sex, age, affected side, time after injury, and motor function were found to be the most common and frequently reported anthropometric variables. However, other important features such as stroke location, severity, ethnicity, pre-existing comorbid conditions (diabetes, cognitive impairment, or depression) [21], and pre-stroke lifestyle factors (social engagement and exercise) were not consistently considered [40]. Also, body function, structure, activity limitations, and participation restriction should be assessed using appropriate and adequate clinical measures. Indeed, although the Fugl–Meyer Assessment (FM) was commonly used in the present findings, it was not included in all studies, which may affect our understanding of participants’ behavior [21]. 

It is important to note that limited or varied demographic and stroke-related data may hinder our understanding of stroke recovery trajectories [41]. Therefore, it is crucial to follow the core recommendations for stroke trials, which state that pre-stroke clinical, demographic, and stroke-related data are core measures that should be collected to improve the clinical prediction of recovery and characterization of patient cohorts. For instance, active hand movements and walking assessment are recommended, especially in studies with chronic stroke, when the NIHSS could not be collected at stroke onset [21].

Another important consideration is the presence or absence of non-disabled matched individuals, which facilitates comparisons. Comparing an individual’s motor task performance with a reference population of non-disabled matched individuals has been shown to be advantageous for better understanding disabled performance [21]. Despite this assumption, this was only considered in about half of the present studies.

### 4.2. Tasks and Non-Linear Measures 

The intrinsic variability of human movement can be assessed by considering two parameters: stability and complexity [9,42]. Both parameters can be measured using several non-linear measures, such as the MLyE, the maxFM, and fractal measures like MFDFA for stability assessment. In addition, entropy measures are used to quantify the irregularity that is associated with movement complexity [7,9,43].

Although entropy measures provide valuable insights into one aspect of variability, they do not capture the structural abundance and widespread component characterization of a complex system operating across multiple spatial and temporal scales [44]. Therefore, it is recommended to combine them with other non-linear measures for a more comprehensive assessment [7]. Entropy was the most used measure for processing kinetic, kinematic, and EMG data in the reviewed studies. It is worth noting that none of the studies combined entropy with other non-linear measures, and only two studies combined different entropy measures [32,37].ApEn is a metric commonly used in the biomechanics literature to assess complexity [9,45]. However, SampEn was the most frequently reported measure in the studies reviewed, followed by ApEn. Despite its frequent use, ApEn may lead to inconsistent results due to its sensitivity to the length of the time series [9]. It should be noted that the studies included in this review used ApEn to quantify the temporal structure of variability during three trials of reaching-to-grasp [27,28] and reaching-to-point [33] tasks. Although the authors normalized the ApEn values to the length of their time series [27,28,33], the number of trials and time series length may not be sufficient for consistent results.

SampEn is generally considered a better option for analyzing time series due to its greater relative consistency and lesser dependence on the length of the dataset [20]. It has been widely used as a complexity measure in various fields of research to evaluate the impact of age, illness [46], and performance [47]. In clinical contexts, this measure can help identify motor coordination disorders [48]. In this review, five of the studies used SampEn, with short-length tasks, to quantify the complexity of the sEMG signal during knee extension [29] and elbow flexion [32,34]; Tang et al. [36] used it as an indicator of the complexity and randomness of the sEMG signal during elbow flexion; and it was used as an indicator of the temporal structure of variability (force regularity) during wrist or finger extension [19]. Additionally, SampEn can differentiate specific characteristics of distinct populations [12]. 

Although SampEn demonstrates relative consistency and less dependence on data length, its similar definition of vectors is based on the Heaviside function, as in ApEn [49]. However, the use of the Heaviside function has inherent flaws, which can lead to problems in the validity of the entropy definition, particularly when small parameters are involved [50]. In this review, two studies used fApEn [30,32] to measure the time series regularity and complexity of sEMG signals of BIC and TRI during an arm tracking task. This measure was developed resulting from a combination of the concept of “fuzzy sets” and ApEn [51], showing better relative consistency and robustness to noisy physiological signals with short data length [52,53].

Because neuromuscular activities are inherently non-linear [54], cross-entropy-based measures are used to characterize pattern synchronization and quantify inter-muscular coupling [37]. Xie et al. [53] introduced the C-FuzzyEn, based on FuzzyEn. This method appears to be better suited to short time series and is more robust to noise, allowing the observation of the global intermuscular coupling between two separated but interconnected sEMG signals [53]. C-FuzzyEn was used in a study included in this review to characterize the global intermuscular coupling during a tracking task of elbow flexion and extension [37]. The iC-FuzzyEn and i-FuzzyEn are proposed by the authors to characterize the instantaneous intermuscular coupling and the dynamic complexity, respectively, in order to gain a better understanding of the neuromuscular mechanisms after a stroke [37].

Van Emmerik and colleagues [9] demonstrated that ApEn, SampEn, and their variants are limited in their ability to provide a detailed analysis of physiological signals because they only assess entropy on a temporal scale. As physiological systems exhibit structures across multiple time scales [55], mMSE was developed to quantify the intrinsic complexity of the system at small and large scales [55,56,57]. Nevertheless, only one of the studies included in this review used it to analyze the complexity of gait after stroke [18]. This may suggest an emerging interest in using measures that can better reflect the impact of complexity on the adaptability of the motor system after stroke.

According to the abovementioned studies, using different entropy measures shows a variety of approaches to the analysis of the data. The term complexity seems to have acquired synonyms such as ‘randomness’, ‘temporal structure of variability’, ‘predictability’, or ‘regularity’, depending on the research question of the studies. Therefore, the variety of entropy measures used in the studies analyzed in our review suggests an increasing interest in measures that can more accurately capture the complexity of movement.

Despite the growth of entropy measures, some authors question their validity in assessing the complexity of motion [42]. With our review, MFDFA [38], MLyE [35], short-term LDE [31] and MaxFM [31] were identified as non-linear measures to assess stability during a treadmill (TM) walking task. These are commonly used to assess the local (MLyE and short-term LDE) and orbital (MaxFM) stability of the neuromuscular system, as in the ability to attenuate small perturbations [43].

Xu et al. [38] considered MFDFA to assess gait variability. This measure allows one to capture the multifractal scaling elements in a nonstationary time series [58]. Fractal patterns are frequently observed in healthy and well-controlled movements, indicating a certain level of adaptability and flexibility in motor control [59], and providing information about the underlying control processes involved in movement [60]. As human gait is a cyclic and repetitive set of movements, fractal analysis can be a valuable tool in assessing gait adaptability and flexibility, identifying gait disorders, and predicting fall risk due to central nervous system dysfunction [39,59,61]. Concerning the quantification of the local dynamic stability of complex non-linear systems, MLyE using Wolf’s algorithm [35] and short-term LDE [31] were the measures applied. MLyE quantifies the rate of trajectory convergence or divergence in an n-dimensional state phase and how sensitive the system is to initial conditions [11,62,63]. Short-term LDE, on the other hand, is a measure of the local divergence or convergence of trajectories over a short period of time [11]. It focuses on how trajectories behave in the immediate neighborhood of a given point in phase space [11]. Hence, both concepts are related to the sensitivity of a dynamic system to initial conditions [64]. However, MLyE is typically concerned with long-term behavior, while short-term LyE focuses on local behavior over shorter time intervals, which is consistent with the included studies [31,35]. In the present review, it was identified that Wolf’s method was used to calculate MLyE [35]. Nevertheless, the algorithm developed by Rosenstein is the most widely used in biomechanical studies [65]. Considering the length of the data in Kempski et al.’s [35] study, Wolf’s algorithm appears to be more appropriate for small gait datasets [66]. The authors of this study considered, based on Moraiti et al. [67], that assessing two minutes of walking is sufficient for accurately calculating the LyE.

The MaxFM method, which quantifies the convergence/divergence of continuous gait variables towards a limit cycle [43], seems to be particularly relevant for analyzing long-distance walking or walking with many steps [68]. However, this condition was not observed in the study that used this measure [35]. In this case, the authors needed to adjust the protocol because the population included (post-stroke) was unable to walk long distances. 

These measures address different aspects of stability and can complement each other, revealing underlying patterns, attractor states, and dynamical properties that are not evident through traditional linear methods.

Although non-linear measures can be helpful in interpreting variability dynamics, it is important not to underestimate the importance of appropriate parameter selection [69]. Inadequate selection of non-linear analysis parameters can impact the measurement of system complexity, leading to inconsistent outcomes [70]. Time series data from biological systems are typically non-stationary and noisy, containing extreme values [71]. Noise can have an impact on both the magnitude and structure of variability, potentially obscuring patterns and leading to inaccurate results [72]. To reduce noise and facilitate the accurate identification and analysis of non-linear features [60], filters were employed in eleven of the studies included, with frequencies that preserve relevant physiological information [18,19,27,28,29,31,32,33,34,36,37]. An important methodological consideration is the length of the data time series, as almost all non-linear measures are affected by this feature [69]. Regarding the specific parameters m, r, N, and lag, most of the included studies did not explain why they were chosen, and no article described all of them at the same time. Lag was described in only three studies written by the same study group [27,28,33]. It is important to note that all authors included the data collection time/number of trials in their evaluation protocol. However, two authors did not specify which data were included in the non-linear analysis [37,38]. To improve clarity, it is recommended to define the specific data used in the analysis. 

Among the tasks analyzed, it is important to highlight that three studies were related to reaching [27,28,33] and four to walking [18,31,35,38], with both being considered functional daily tasks. The remaining protocols studied focused on isolated components of task execution, such as muscular strength [19,29,30,34,36], muscle activity [30,32,34,36,37], or range of motion [32]. However, research in movement sciences suggests that understanding integrated measures of task execution, such as coordination and adaptability, can provide the necessary information for the mechanisms underlying human movement [73]. Therefore, an incomplete assessment limited to the execution of isolated components diminishes the understanding of movement dysfunction [73]. The study of SMH should involve analyzing various motor tasks [74]. Although the reviewed studies analyzed several tasks (reaching and walking), it is important to also consider other core tasks such as sitting, standing, transitioning from sitting to standing, transitioning from standing to sitting and climbing or descending a step [75]. These tasks can be used as a starting point for different clinical situations and can help to identify critical aspects of movement [75,76]. This may lead to the development of a standardized approach for analyzing human movement [75,76].

Considering the walking tasks, variability has been assessed in both overground (OG) [18] and TM walking [31,35,38]. The use of TM walking emphasizes the need for a significant amount of continuous data to calculate all non-linear dynamic measures, particularly LyE [77]. However, when using a TM, the constant speed imposes constraints on the gait, reducing the amount of movement variability. It appears to make the temporal structure of gait variability unrealistically regular compared to OG walking [78,79]. Sloot et al. [80] suggested that multiple episodes of shorter data can also be used to increase statistical precision. Therefore, OG walking appears to be a more accurate reflection of gait variability outcomes.

Another aspect to emphasize is that around nine of the included studies focused on the assessment of UL, rather than LL. It has been reported in the literature that the LL recovers more rapidly than the UL [81,82,83,84,85], and that about 40–50% of stroke survivors have UL dysfunction [86]. Considering this review in a chronic stroke population, the results obtained may reflect these assumptions. Also, the lower number of results with protocols involving LL tasks seems to reduce the fact that LL and gait may not express the higher CNS control centers. Thus, walking in humans is mainly produced by the combined roles of the reflex circuit, which produces motor patterns triggered by sensory feedback, and the central pattern generators (CPGs) [87,88]. The CPGs mainly innervate the muscles of the lower limb [89], and they may not be affected following a stroke [90]. Consequently, rhythmic pattern movement such as stepping during walking can be generated even in the absence of control of the higher CNS areas [87]. However, further investigation and analysis are needed to validate this hypothesis and understand the complex mechanisms underlying motor control in the lower limbs.

Incorporating the principles of variability and chaotic variation, together with non-linear tools, can provide additional insights to guide practice and assess human movement dysfunction. It is important to note that non-linear analysis complements traditional linear methods in the understanding of neuromotor control following central nervous system dysfunction.

### 4.3. Assessment Instruments and Kinematic, Kinetic and EMG Variables

In terms of kinematic data analysis instruments, three motion capture systems were used, namely VICON™ [27,28,33], Motion Analysis Corporation [31,35], and OptiTrack^®^ [32,38], mostly for upper limb tasks. These systems feature high-precision optoelectronic cameras that are considered highly accurate measurement tools for kinematic analysis in the study of human movement [91]. In one of the included studies [18], inertial measurement units (IMUs) were used to evaluate kinematic data during walking. Despite the high accuracy and precision of motion capture systems, they are relatively costly and time-consuming and only allow data to be evaluated in a laboratory context [92]. IMUs can collect data outside the laboratory environment [93] to assess human movement in an easier, low-cost, valid, and reliable way [94,95]. Thus, in the context of research or clinical practice, they could be a viable alternative to motion capture systems. They allow evaluation in a simulated context, as described in the articles included in this review, but also over extended periods in real-world environments [73]. IMUs could contribute to the improvement of motor variability assessment by providing essential data required for the interpretation of real-world readiness for participation [73].

The accuracy of motion capture systems depends on the relative positions of the cameras, the position, the type and number of markers, and their movement within the capture volume [96]. The number of cameras in the included studies ranged from 6 [38] to 12 [27,28,33]. The use of a larger number of cameras can be seen as an advantage, knowing that more cameras allow for better visual coverage [97], help to avoid marker occlusion by body parts, and allow for better reconstruction of motion in the observed time frame [98].

The optoelectronic systems mentioned in the included studies [27,28,31,32,33,35,38], used passive marker systems that reflect light back to the sensor. Although the active marker systems may provide more robust measurements, they require additional cables and batteries, limiting freedom of movement [98]. The passive marker systems appear to be less invasive and can provide highly accurate information and simultaneous 3D kinematic measurements during different tasks.

Different motion capture protocols have been found, with varying marker sets, positions, and numbers. The well-documented and consistent use of a set of 67 reflective markers for upper limb joint kinematics is found in only three studies [27,28,33]. Kinematic results are highly sensitive to anatomical markers [99,100]. The use of different marker sets can be a source of variability that can have a significant impact on the kinematic data generated [101]. The only study that used IMUs placed on the LL and pelvis did not specify the number and exact placement of the sensors. Sensor specifications, such as differences in sensor range, sampling rate, and placement, may be the reasons for variability in the reporting of non-linear measurements [102].

Furthermore, the lack of a standardized procedure for kinematic analysis may lead to discrepancies between studies. Data collection, processing, and analysis should be standardized and properly reported to facilitate comparisons between studies and the establishment of reference values for the field. The actual accuracy and precision achieved in practice may vary depending on the setup, user expertise, and environmental conditions [103]. Therefore, appropriate models for the specific research purpose, a single investigator to accurately place the markers [99] or sensors, prior testing of the experimental protocol, and improvement of the most accurate setups could be important factors in increasing the reliability of the data.

A wide variety of kinematic results were observed, with the most pronounced being those related to the joint kinematics of the upper [27,28,32,33] and lower limbs [35,38]. Kinematic assessment is a reliable and objective method and provides quantitative measures of movement control, including motor performance and movement quality [103,104]. Only two authors suggested combining joint kinematics with spatial–temporal parameters [33,35]. This approach integrates the complexity of joint motion and overall movement patterns in space and time, promoting objectivity and practicality by ensuring that assessments are directly related to real-world activities and functional tasks [105]. 

Regarding the kinetic data, the analyzed articles assessed the torque of several muscles. The UL torque muscle was the most assessed, using an isokinetic dynamometer [30], a torque sensor [34], or a force transducer [19]. An isokinetic dynamometer is frequently used in scientific research to assess muscle strength due to its simplicity and accuracy [106]. It is a reference in the comparison of measurements obtained with other instruments, with excellent levels of validity [107,108] and reliability of muscle strength assessment in UL and LL in chronic post-stroke patients [106,109,110,111].

The use of sEMG systems has been prevalent in assessing muscle activity during UL-isolated components of task execution [30,32,34,36,37]. Clinically, sEMG provides a non-invasive method to observe neuromotor activity and to assess the individual’s neurological status and nervous system reorganization [112]. 

However, neuromuscular signals have limitations in providing insights into movement quality, which is often assessed via movement function tests or biomechanical performance [113]. Additionally, a broader range of assessment methods and metrics that integrate the benefits of biomechanical (kinematic and kinetic) and neuromuscular (sEMG) measures have been highlighted [113,114,115]. Although one study combined kinematic and sEMG data [32] and two studies combined kinetic and sEMG data [30,34], none of the others attempted this. Combining data from different domains can provide comprehensive insights by enabling the understanding of the origin of neuromuscular activity through the expression of biomechanical variables. The complexity of human movement’s neuromotor behavior justifies a combined approach. Particularly in cases of neurological injury, kinematic and kinetic changes occur due to an altered ability to influence neural circuits, whose function is intrinsically linked to muscle activity [116]. Such changes can be more accurately documented using sEMG [117].

Some limitations need to be acknowledged. Firstly, the search was restricted to five databases, which means that it is possible that we may have overlooked some of the literature. Secondly, we intentionally developed a broad search strategy to prevent the possibility of missing important studies. Nevertheless, this scoping review and its findings seem to be indicative of the advances made in this field. The study of non-linear dynamics will allow us to understand the complexity of human movement and evaluate their biological adaptivity.

## 5. Conclusions

This scoping review provides a comprehensive overview of the current research landscape on non-linear measures in the analysis of human movement post-stroke. Entropy measures, particularly sample entropy, have been the preferred measures when investigating the complexity of human movement. The focus has been on UL tasks such as reaching and components of task execution, such as elbow flexion and extension. Regarding the analyzed variables, the joint kinematics and muscle torque and activity were the most prominent during the UL tasks. Assessment instruments employing motion capture systems and sEMG were the most used tools for kinematic and muscle activity analysis, respectively.

## Figures and Tables

**Table 1:** Eligibility criteria according to PCC.

Criteria	
Population	Chronic [24] poststroke adults (>19 years old)
Concept	Non-linear measures in kinetic, kinematic, or EMG data processing of human movement analysis
Context	Open

**Table 2:** Characteristics of the participants, study design, and tasks.

Author, Year	Study Design	Participants	Task
Sethi et al., 2013 [27]	Observational, analytical study (with healthy control group)	n = 16 (11 M/5 F) 67.60 ± 8.1 years old 6 CLR/10 CLL 16 Isch 71.31 ± 48.84 months poststroke UE_FM 37.27 ±9.27	Performed three valid reaching trials to grasp a soda can (56 mm diameter; 208 mm circumference) placed at 80% arm’s length on a table in front of each shoulder with the paretic UL. All participants wore dark sleeveless shirts and sat on an adjustable backless bench with knees bent at 90° and feet flat on the floor. Their hands were placed palms down on a table in front of them and supported at 90° elbow flexion by armrests positioned flush with the table. They were instructed to reach for the can, lift it off the table, place it back on the table, and return to the starting position as quickly as possible.
Sethi et al., 2013 [28]	Quasi-experimental	n = 6 (2 M/4 F) 67.00 ± 10.69 years old 2 CLR/4 CLL 6 Isch 41.83 ± 35.01 months poststroke UE_FM 32 ± 6.9 MAS < 2 MAL ≤ 3	Performed three valid reaching trials to grasp a soda can (56 mm diameter; 208 mm circumference) placed at 80% arm’s length on a table in front of each shoulder with the paretic UL. All participants wore dark sleeveless shirts and sat on an adjustable backless bench with knees bent at 90° and feet flat on the floor. Their hands were placed palms down on a table in front of them and supported at 90° elbow flexion by armrests positioned flush with the table. They were instructed to reach for the can, lift it off the table, place it back on the table, and return to the starting position as quickly as possible.
Chow and Stokic, 2014 [29]	Randomized Controlled Trial (with healthy control group)	n = 23 (13 M/10 F) 64.90 ± 13.6 years old 9 CLR/14 CLL 16.6 ± 22.7 months poststroke LE_FM 26.0 ± 6.7 MAS < 2 RMI 13.4 ± 6.7	Performed 2 trials of maximum isometric knee extension (10 s) of both legs, in random order, at different levels (10%, 20%, 30%, or 50% of MVC), as accurately and consistently as possible, in a seated position, with 60 s of rest in between. Subjects were asked to extend the knee and match the displayed torque signal (a horizontal line) to a specified target force, marked on the monitor and placed according to individual preference, usually 40–50 cm in front of the head.
Sun et al., 2014 [30]	Quasi-experimental	n = 8 (7 M/1 F) 50.13 ± 9.13 years old 3 CLR/5 CLL 4 Hemo / 4 Isch 132.00 ± 101.76 months poststroke	Two tasks were carried out: Performance of the MIVE and the MIVF on the affected elbow at 90° of flexion, 3 times for 5 s each. Performing 3 repetitive arm tracking trials, starting with the elbow at 90°. Two points were displayed in real-time on a computer screen in front of the subjects. Subjects attempted to control their elbow movement to track and match the target pointer. During the elbow extension, the robotic system continuously generates an assistive torque to support the elbow movement.
Kao et al., 2014 [31]	Observational, analytical study (with healthy control group)	n = 9 (5 M/4 F) 60.8 ± 9.0 years old 40.8 ± 39.6 months poststroke LE_FM 27 ± 4	Walking on a treadmill at 60%, 80%, and 100% of the preferred walking speed (determined from 5 trials of walking 10 m on the ground) and the fastest achievable speed (determined as the fastest speed the subjects could maintain for 1 min). Each speed was tested 3 times for 1 min.
Ao et al., 2015 [32]	Observational, analytical study (with healthy control group)	n = 11 (9 M/2 F) 47.00 ± 10.86 years old 4 CLR/7 CLL 4 Hemo/7 Isch 53.30 ± 50.9 months poststroke MAS ≤ 3	Performed 18 trials (36 s each) of elbow flexion, divided into 3 blocks (at 6 different speed levels), in a sinusoidal trajectory tracking task ranging from 30° to 90°, with 5 min of rest between each block and 30 s of rest between each trial. Subjects were seated at a table with the elbow at 30° of flexion and the shoulder at almost 90° of abduction, with the forearms attached to a lightweight aluminum manipulandum. Target and elbow angles were provided by real-time feedback on a computer screen placed in front of the subjects.
Sethi et. al., 2017 [33]	Observational, analytical study	n = 10 (9 M/1 F) 67.00 ± 8.90 years old 4 CLR/6 CLL 10 Isch (3 RMCA, 2 LMCA, 1 PVWM, 1 LLI, 1 LPI, 1 RCS, 1 RSI) 53.30 ± 50.9 months poststroke UE_FM 36.9 ± 7.9	Performed 3 trials of reach-to-point movements (drawn at 80% arm length) in three conditions, without randomization: - Voluntary reaching at preferred speed (PREFERRED). - Reaching as fast as possible (FAST). - Reaching with rhythmic auditory cues generated by a metronome (RHYTHM). Participants were seated on an adjustable backless bench with no trunk restraint, knees bent at 90°, and feet flat on the floor. Their hands were placed palms down on a table in front of them and supported at 90° elbow flexion by armrests level with the table. Post-stroke participants reached the target with a more impaired UL.
Zhang et al., 2017 [34]	Observational, analytical study	n = 10 (7 M/3 F) 62.40 ± 12.34 years old 5 CLR/5 CLL 49.20 ± 26.47 months poststroke MAS < 2	Performance of two MVC trials of submaximal isometric elbow flexion for 10 s each at different torque targets (10% to 80%), with the impaired and non-impaired arm separately. The target values were displayed as visual targets on the computer screen in a random order. During the period of isometric muscle contraction, which lasted from 2 s to 8 s, the subjects were verbally instructed to match the visual target and to maintain it as accurately as possible for all trials. Subjects were seated comfortably in a height-adjusted chair with the arm to be tested firmly attached to a custom-made device with the shoulder flexed approximately 30° and abducted 45°, the elbow flexed 90°, and the forearm in a neutral position. The other arm was placed next to the body.
Kempski et al., 2018 [35]	Observational, analytical study	n = 7 (5 M/2 F) 62.57 ± 5.86 years old 4 CLR/3 CLL 42.30 ± 49.03 months poststroke LE_FM 25.71 ± 5.22	Walking in sports shoes for 2 min on an instrumented split-belt treadmill at an individual’s self-selected speed (determined by a 10 m walk test) and long-distance walking speed (determined by a 6 min walk test).
Tang et. al., 2018 [36]	Observational, analytical study (with healthy control group)	n = 11 (7 M/4 F) 61.55 ± 12.05 years old 6 CLR / 5 CLL 46.00 ± 27.00 months poststroke UE_FM 43.14 ± 11.68 MAS < 2	Performed 3 trials of elbow flexion with isometric graded increasing force levels corresponding to 10%, 30%, 50%, 70%, submaximal (90%), and almost 100% of MVC for at least 3 s each. Sufficient rest was allowed between 2 consecutive trials to avoid mental or muscular fatigue. Subjects were seated comfortably in a mobile chair or wheelchair next to a height-adjustable desk, with the tested elbow flexed at 90 degrees on the paretic and contralateral side in a randomized order.
Dugan et. Al., 2020 [18]	Observational, analytical study (with healthy control group)	n = 11 (8 M/3 F) 57.91 ± 14.7 years old 177.34 ± 7.2 cm LE_FM 25.00 ± 2.93	Walking for 2 min at their preferred speed around a level, tiled 60 m indoor walking track, without any aids or LL orthoses.
Kim et al., 2020 [19]	Observational, analytical study	n = 14 (7 M/7 F) 63.80 ± 15.9 years old 4 CLR/10 CLL 2 Hemo / 12 Isch 38.7 ± 45.2 months poststroke Hand Function (%) in SIS (3.0) 52.9 ± 31.7 MMSE > 23	Performance of 2 isometric force control tasks in 3 different randomized conditions: unimanual paretic, unimanual non-paretic, and bimanual. - 2 Submaximal force control task of 3 MVC trials (5 s) with a rest interval of 60 s - 2 Maximal sustained force production task of 3 MVC trials (8 s) with a rest interval of 60 s. For both tasks, participants performed either isometric unimanual or bimanual wrist and finger extension upwards (lifting) towards the padded platforms. Participants sat 78 cm away from a 43.2 cm LCD monitor and placed their forearms on the table in a stable position, maintaining 15–20° of shoulder flexion and 20–40° of elbow flexion. They were instructed to place either unilateral or bilateral hands under the specially padded platforms and to adjust the height of the platform.
Tian et al., 2021 [37]	Observational, analytical study (with healthy control group)	n = 3 (2 M/1 F) 52.33 ± 24.19 years old 1 CLR/2 CLL 2 Hemo/1 Isch 12.67 ± 5.03 months poststroke UE_FM 33.42 ± 19.70 MAS < 2 MMSE > 23	Two tracking tasks of elbow flexion and extension were performed in which the activation of the agonist muscle increased from 0% to 15% MVC in 0–5 s, then gradually relaxed within 5–10 s and returned to 0. Each round movement was performed at a constant speed (15%MVC/5 s) from the origin to the target point, and these included pointing (0% to 15% amplitude) under elbow flexion and pointing (15% to 0% amplitude) under elbow extension. The above procedure was repeated 3 times as a trial. Subjects randomly performed 10 trials (5 flexion trials and 5 extension trials) and rested for 30 s after each trial. During the performance, they were instructed to sit in a height-adjusted chair and place their impaired arm on the horizontal armrest with the elbow flexed at 90° and the shoulders abducted at 90°. They were asked to grasp the handle attached to the armrest.
Xu et. al., 2022 [38]	Observational, analytical study (with healthy control group)	n = 10 (8 M/2 F) 48.3 ± 12.8 years old	Performed 3 trials of walking on a treadmill at a comfortable speed for 3 min, with 5 min of rest in between, using special experimental shoes.

M: male; F: female; CLR: contralesional right; CCL: contralesional left; Isch: ischemic; Hemo: hemorrhagic; UE_FM: Fugl–Meyer Assessment Upper Extremity; LE_FM: Fugl–Meyer Assessment Lower Extremity; MAS: Modified Ashworth Scale; MAL: Motor Activity Log; RMI: Rivermead Mobility Index; SIS: Stroke Impact Scale; MMSE: Mini Mental State Examination; MVC: maximal voluntary contraction; MIVE: maximum isometric voluntary extension. MIVF: maximum isometric voluntary flexion; UL: upper limb; LL: lower limb.

**Table 3:** Assessment instruments, kinematic, kinetic, and EMG variables, non-linear measures, and their parameters.

Author, Year	Assessment Instrument	Kinematic, Kinetic, and EMG Variables	Non-Linear Measure	Non-Linear Measures Parameters
Sethi et al., 2013 [27]	Kinematics: ▪ Twelve-camera VICON™ motion capture system (Vicon 612; Oxford Metrics Inc., Oxford, UK) ▪ 67 reflective markers in UB were placed using a marker set described by the VICON Plug-In-UE.	Joint Kinematics: ▪ Shoulder, elbow, wrist, and PIP index finger (°)	▪ ApEn	▪ Kinematic data not filtered and surrogation with Theiler’s first algorithm (20 surrogate time series of each trial) ▪ Each joint angle time series was analyzed from the start of the reach through the entire length of the respective time series including the pauses between the 3 trials. ▪ 4 time series were obtained (one for each joint) ▪ lag = 1, m = 2, and r = 0.2 times the SD of the time series ▪ ApEn values of each participant were normalized to the length of their time series
Sethi et al., 2013 [28]	Kinematics: ▪ Twelve-camera VICON™ motion capture system (Vicon 612; Oxford Metrics Inc., Oxford, UK) ▪ 67 reflective markers in UB were placed using a marker set described by the VICON Plug-In-UE.	Joint Kinematics: ▪ Shoulder, elbow, and wrist (°)	▪ ApEn	▪ Kinematic data not filtered and surrogation with Theiler’s first algorithm (20 surrogate time series of each trial) ▪ Each joint angle time series was analyzed from the start of the reach through the entire length of the respective time series including the pauses between the three trials. ▪ 4 time series were obtained (one for each joint) ▪ lag = 1, m = 2, and r = 0.2 times the SD of the time series ▪ ApEn values of each participants were normalized to the length of their time series
Chow and Stokic, 2014 [29]	Kinetics: ▪ Biodex System 3 isokinetic dynamometer (Biodex Medical Systems, Inc., New York, NY, USA) and a custom-built amplifier connected directly to the torque sensor of the dynamometer (overall sensitivity 57.5 mV/Nm)	Torque Measurement: ▪ MVC torque of quadriceps (Nm) ▪ MVC Power of quadriceps (Nm2)	▪ SampEn	▪ Torques were filtered using a second-order Butterworth low-pass filter with a 30-Hz cutoff ▪ Only the middle 8 s of each 10 s trial were analyzed. Out of 2 trials collected at each force level, the one with a lower CV was used for statistical analysis. ▪ N = 800, m = 3, and r = 0.2 SD of the time series
Sun et al., 2014 [30]	Kinetics: ▪ Robot-aided developed dynamometer electromyography: ▪ sEMG recording system (Noraxon, Scottsdale, AZ, USA)	Torque Measurement: ▪ MIVE torque of TRI (Nm) ▪ MIVF torque of BIC (Nm) Electromyographic activity: ▪ Raw EMG data of TRI and BIC	▪ fApEn	▪ The EMG data were not filtered ▪ N = 1000, m = 2, and r = 0.2 SD of the time series Torque Measurement: ▪ A segment of 1000 samples (500 samples before and after the maximum value) was selected for calculating fApEn Electromyographic activity: fApEn values were calculated with a 1000 ms sliding window and a 10 ms window increment in consideration of the computation power of the computer
Kao et al., 2014 [31]	Kinematics: ▪ Eight-camera video system (Motion Analysis Corporation, Santa Rosa, CA, USA) ▪ 46 reflective markers attached on the LB, trunk, and over the C7 vertebra	Spatiotemporal parameters: ▪ Vertical, anteroposterior and mediolateral of C7 velocities (m/s)	▪ Short-term LDE ▪ maxFM	▪ Delay-embedded state spaces were reconstructed independently from the 3 velocities of non-filtered C7 vertebral marker data ▪ Data from 30 continuous strides were extracted for each trial ▪ Short-term LDE: ▪ Data were resampled to 3000 total data points, approximately 100 data points per stride maxFM: ▪ The state spaces were first divided for individual strides and then each stride was time normalized to 101 samples
Ao et al., 2015 [32]	Kinematics: ▪ Motion capture system (OptiTrack^®^, NaturalPoint, USA) ▪ Two reflective markers are attached to the elbow joint and at the end of the handle Electromyography: ▪ Tele-EMG system (MyoSystem2400T, Noraxon, USA) ▪ 2 Ag/AgCl bipolar surface electrodes (Noraxon, USA)	Joint Kinematics: ▪ Elbow (°) Electromyographic activity: ▪ EMG Amplitude (mv) TRI and BIC	▪ fApEn ▪ ApEn ▪ SampEn	▪ The EMG signals were band-pass filtered through a 4th-order, zero-phase Butterworth digital filter with a frequency band from 5 to 400 Hz and a 50 Hz digital notch filter ▪ The performances of the three entropies were compared using both simulated signals MIX(p) and real EMG signals. Three simulated signals, MIX(0.1), MIX(0.5) and MIX(0.9) were generated and each one had 5000 samples ▪ The 100-point segments were selected randomly from each of the 5000-sample simulated MIX(0.1), MIX(0.5), and MIX(0.9) signals, respectively ▪ m = 2 ▪ N and r were carried out to find a suitable complexity measurement with optimal N and r
Sethi et. al., 2017 [33]	Kinematics: ▪ Twelve-camera VICON™ motion capture system cameras (Vicon 612/T40; Oxford Metrics, Oxford, England) ▪ Sixty-seven reflective markers in UB were placed using a marker set described by the VICON Plug-In-UE	Joint Kinematics: ▪ Shoulder, elbow, wrist, and PIP index finger (°) Kinematics Spatiotemporal Parameters: ▪ Index finger velocity (m/s) ▪ PV (m/s)	▪ ApEn	▪ Kinematic data not filtered and surrogation with Theiler’s first algorithm (20 surrogate time series of each trial) ▪ Each joint angle time series was analyzed from the start of the reach through the entire length of the respective time series including the pauses between the three trials. ▪ 4 time series were obtained (one for each joint) ▪ lag = 1, m = 2, and r = 0.2 times the SD of the time series ▪ ApEn values of each participant were normalized to the length of their time series
Zhang et al., 2017 [34]	Kinetic: ▪ One torque sensor (Model TRS 500, Transducers Techniques, CA) on BIC Electromyography: ▪ Porti sEMG system (TMS International, The Netherlands)	Torque Measurement: ▪ MVC torque of elbow flexion (Nm) Electromyographic activity: ▪ Root mean square (V) BIC	▪ SampEn	▪ The EMG signals were filtered using a zero-lag band-pass (20–450 Hz) fourth-order Butterworth filter ▪ The torque signal was filtered using a zero-lag low-pass second-order Butterworth filter ▪ Both surface EMG and torque data correspond to the same 2 s time window selected for data analysis. ▪ N = 4000, m = 2, and r = 0.25 times the SD of the time series
Kempski et al., 2018 [35]	Kinematics: ▪ Eight-camera motion capture system (Motion Analysis Corporation) ▪ Twenty-five single reflective markers and nineteen markers on shells placed on the bony landmarks and tracking segments	Joint Kinematics: ▪ Hip, knee and ankle (°) Kinematics Spatiotemporal Parameters: ▪ Gait velocity (m/s)	▪ MLyE (Wolf’s algorithm)	▪ Not specified
Tang et. al., 2018 [36]	Electromyography: ▪ Home-made multi-channel sEMG recording system ▪ One large round reference electrode (Dermatrode; American Imex, Irvine, CA) was placed on the arm fossa cubitalis	Electromyography activity: ▪ EMG Integral (V) of BIC	▪ SampEn	▪ The EMG signal was filtered with a fourth-order zero-lag non-causal Butterworth band-pass filter set at 20–500 Hz. Then, a set of second-order notch filters were used to remove the 50 Hz power line interference and its harmonics. ▪ N: the EMG signal was divided into several non-overlapping epochs, each with a time length of 1 s (equivalent to 1000 sample points) ▪ m = 2 and r = 0.25 times the SD of the time series
Dugan et. al., 2020 [18]	Kinematics: ▪ Wireless Inertial Measurement Units (IMUs) (InterSense Inertia Cube BT) attached to each LL segment and pelvis	Joint Kinematics: ▪ Hip, knee and ankle (°)	▪ mMSE	▪ The tri-axial accelerometer data from each IMU dataset were unfiltered ▪ N: the last 6000 data points, corresponding to the last 100 s of the walking trials were used for analysis. ▪ m = 2 and r = 0.2
Kim et al., 2020 [19]	Kinetics: ▪ Force transducers (MLP-75, Transducer Techniques, 4.16 × 1.27 ×1.90 cm, range = 75 lbs., 0.1% sensitivity) attached to the padded platforms	Force Measurement: ▪ MVC torque of wrist and finger extension (N)	▪ SampEn	▪ The force data were filtered using a bidirectional fourth-order Butterworth filter with a cutoff frequency = 30 Hz ▪ N: for the submaximal force control tasks, the middle 16 s of the force signals were used ▪ N: For the maximal sustained force production tasks, the middle 5 s of the force data were analyzed ▪ m = 2, and r = 0.2 SD of the force signals
Tian et al., 2021 [37]	Electromyography: ▪ sEMG system (DAQ USB- 6341, National Instruments, USA)	Electromyography activity: ▪ Raw EMG data (V) BIC and TRI	▪ C-FuzzyEn ▪ i-FuzzyEn ▪ iC-FuzzyEn	The raw EMG signals were bandpass filtered using a 4th-order Butterworth filter at 20–300 Hz and a 50 Hz notch filter C-FuzzyEn and iC-FuzzyEn: ▪ N = 500 (0,5 s), m = 2, and r = 0.2 SD of the EMG signal i-FuzzyEn: ▪ N = 500, m = 2, and r = 0.15 SD of the EMG signal
Xu et. al., 2022 [38]	Kinematics: ▪ Six-camera motion capture system (OptiTrack^®^, NaturalPoint, USA) ▪ Ten 12 mm reflective markers bilaterally in LL were placed on the second and third metatarsal space, the lateral malleolus, the midleg, the lateral knee, and the mid-thigh	Joint Kinematics: ▪ Knee (°) ▪ Ankle (°)	▪ MFDFA	▪ Proper polynomial order = 2 ▪ The short-scale exponent was computed within the segment size ranging from 20 < n < 100 sample. Meanwhile, the long-scale exponent was evaluated in the range of 170 < n < 1000 sample

UB: upper body; UE: upper extremity; LL: lower limb; sEMG: surface electromyography; PIP: proximal interphalangeal; PV: peak velocity; MVC: maximal voluntary contraction; MIVE: maximum isometric voluntary extension. MIVF: maximum isometric voluntary flexion; BIC: biceps brachii; TRI: triceps; SD: standard deviation; V: volts.

## Data Availability

Not applicable.

## Supplementary Materials

The following supporting information can be downloaded at: https://www.mdpi.com/article/10.3390/s24072338/s1.

## References

[1] Saladin L., Voight M. (2017). Introduction to the movement system as the foundation for physical therapist practice education and research. Int. J. Sports Phys. Ther..

[2] Cavanaugh J.T., Kelty-Stephen D.G., Stergiou N. (2017). Multifractality, Interactivity, and the Adaptive Capacity of the Human Movement System: A Perspective for Advancing the Conceptual Basis of Neurologic Physical Therapy. J. Neurol. Phys. Ther..

[3] Ihlen E.A.F., van Schooten K.S., Bruijn S.M., van Dieën J.H., Vereijken B., Helbostad J.L., Pijnappels M. (2018). Improved Prediction of Falls in Community-Dwelling Older Adults through Phase-Dependent Entropy of Daily-Life Walking. Front. Aging Neurosci..

[4] Stergiou N., Decker L.M. (2011). Human movement variability, nonlinear dynamics, and pathology: Is there a connection?. Hum. Mov. Sci..

[5] Bisi M.C., Stagni R. (2018). Changes of human movement complexity during maturation: Quantitative assessment using multiscale entropy. Comput. Methods Biomech. Biomed. Eng..

[6] da Costa C.S., Batistão M.V., Rocha N.A. (2013). Quality and structure of variability in children during motor development: A systematic review. Res. Dev. Disabil..

[7] Harbourne R.T., Stergiou N. (2009). Movement variability and the use of nonlinear tools: Principles to guide physical therapist practice. Phys. Ther..

[8] Barela A.M.F., Duarte M. (2011). Use of force plate for acquisition of kinetic data during human gait. Braz. J. Mot. Behav..

[9] van Emmerik R.E.A., Ducharme S.W., Amado A.C., Hamill J. (2016). Comparing dynamical systems concepts and techniques for biomechanical analysis. J. Sport Health Sci..

[10] Gorniak S.L. (2019). The relationship between task difficulty and motor performance complexity. Atten. Percept. Psychophys..

[11] Stergiou N. (2016). Nonlinear Analysis for Human Movement Variability.

[12] Montesinos L., Castaldo R., Pecchia L. (2018). On the use of approximate entropy and sample entropy with centre of pressure time-series. J. Neuroeng. Rehabil..

[13] Kędziorek J., Błażkiewicz M. (2020). Nonlinear Measures to Evaluate Upright Postural Stability: A Systematic Review. Entropy.

[14] Feigin V.L., Stark B.A., Johnson C.O., Roth G.A., Bisignano C., Abady G.G., Abbasifard M., Abbasi-Kangevari M., Abd-Allah F., Abedi V. (2021). Global, regional, and national burden of stroke and its risk factors, 1990–2019: A systematic analysis for the Global Burden of Disease Study 2019. Lancet Neurol..

[15] Hartman-Maeir A., Soroker N., Ring H., Avni N., Katz N. (2007). Activities, participation and satisfaction one-year post stroke. Disabil. Rehabil..

[16] Adegoke B.O., Olaniyi O., Akosile C.O. (2012). Weight bearing asymmetry and functional ambulation performance in stroke survivors. Glob. J. Health Sci..

[17] Strongman C., Morrison A. (2020). A scoping review of non-linear analysis approaches measuring variability in gait due to lower body injury or dysfunction. Hum. Mov. Sci..

[18] Dugan E.L., Combs-Miller S.A. (2019). Physiological complexity of gait is decreased in individuals with chronic stroke. Comput. Methods Biomech. Biomed. Eng..

[19] Kim H.J., Kang N., Cauraugh J.H. (2020). Transient changes in paretic and non-paretic isometric force control during bimanual submaximal and maximal contractions. J. Neuroeng. Rehabil..

[20] Liu X., Jiang A., Xu N., Xue J. (2016). Increment Entropy as a Measure of Complexity for Time Series. Entropy.

[21] Kwakkel G., Lannin N.A., Borschmann K., English C., Ali M., Churilov L., Saposnik G., Winstein C., van Wegen E.E., Wolf S.L. (2017). Standardized measurement of sensorimotor recovery in stroke trials: Consensus-based core recommendations from the Stroke Recovery and Rehabilitation Roundtable. Int. J. Stroke.

[22] Tricco A.C., Lillie E., Zarin W., O’Brien K.K., Colquhoun H., Levac D., Moher D., Peters M.D.J., Horsley T., Weeks L. (2018). PRISMA Extension for Scoping Reviews (PRISMA-ScR): Checklist and Explanation. Ann. Intern. Med..

[23] Peters M.D.J., Marnie C., Tricco A.C., Pollock D., Munn Z., Alexander L., McInerney P., Godfrey C.M., Khalil H. (2020). Updated methodological guidance for the conduct of scoping reviews. JBI Evid. Synth..

[24] Bernhardt J., Hayward K.S., Kwakkel G., Ward N.S., Wolf S.L., Borschmann K., Krakauer J.W., Boyd L.A., Carmichael S.T., Corbett D. (2017). Agreed Definitions and a Shared Vision for New Standards in Stroke Recovery Research: The Stroke Recovery and Rehabilitation Roundtable Taskforce. Neurorehabil. Neural Repair..

[25] Aromataris E., Munn Z. (2020). Furthering the science of evidence synthesis with a mix of methods. JBI Evid. Synth..

[26] Page M.J., Moher D., Bossuyt P.M., Boutron I., Hoffmann T.C., Mulrow C.D., Shamseer L., Tetzlaff J.M., Akl E.A., Brennan S.E. (2021). PRISMA 2020 explanation and elaboration: Updated guidance and exemplars for reporting systematic reviews. BMJ.

[27] Sethi A., Patterson T., McGuirk T., Patten C., Richards L.G., Stergiou N. (2013). Temporal structure of variability decreases in upper extremity movements post stroke. Clin. Biomech..

[28] Sethi A., Davis S., McGuirk T., Patterson T.S., Richards L.G. (2013). Effect of intense functional task training upon temporal structure of variability of upper extremity post stroke. J. Hand Ther..

[29] Chow J.W., Stokic D.S. (2014). Variability, frequency composition, and complexity of submaximal isometric knee extension force from subacute to chronic stroke. Neuroscience.

[30] Sun R., Song R., Tong K.Y. (2014). Complexity analysis of EMG signals for patients after stroke during robot-aided rehabilitation training using fuzzy approximate entropy. IEEE Trans. Neural Syst. Rehabil. Eng..

[31] Kao P.C., Dingwell J.B., Higginson J.S., Binder-Macleod S. (2014). Dynamic instability during post-stroke hemiparetic walking. Gait Posture.

[32] Ao D., Sun R., Tong K.Y., Song R. (2015). Characterization of stroke- and aging-related changes in the complexity of EMG signals during tracking tasks. Ann. Biomed. Eng..

[33] Sethi A., Stergiou N., Patterson T.S., Patten C., Richards L.G. (2017). Speed and Rhythm Affect Temporal Structure of Variability in Reaching Poststroke: A Pilot Study. J. Mot. Behav..

[34] Zhang X., Wang D., Yu Z., Chen X., Li S., Zhou P. (2017). EMG-Torque Relation in Chronic Stroke: A Novel EMG Complexity Representation with a Linear Electrode Array. IEEE J. Biomed. Health Inform..

[35] Kempski K., Awad L.N., Buchanan T.S., Higginson J.S., Knarr B.A. (2018). Dynamic structure of lower limb joint angles during walking post-stroke. J. Biomech..

[36] Tang X., Zhang X., Gao X., Chen X., Zhou P. (2018). A Novel Interpretation of Sample Entropy in Surface Electromyographic Examination of Complex Neuromuscular Alternations in Subacute and Chronic Stroke. IEEE Trans. Neural Syst. Rehabil. Eng..

[37] Tian N., Chen Y., Sun W., Liu H., Wang X., Yan T., Song R. (2021). Investigating the Stroke- and Aging-Related Changes in Global and Instantaneous Intermuscular Coupling Using Cross-Fuzzy Entropy. IEEE Trans. Neural Syst. Rehabil. Eng..

[38] Xu P., Yu H., Wang X., Song R. (2022). Characterizing stroke-induced changes in the variability of lower limb kinematics using multifractal detrended fluctuation analysis. Front. Neurol..

[39] Amirpourabasi A., Lamb S.E., Chow J.Y., Williams G.K.R. (2022). Nonlinear Dynamic Measures of Walking in Healthy Older Adults: A Systematic Scoping Review. Sensors.

[40] Pongmoragot J., Lee D.S., Park T.H., Fang J., Austin P.C., Saposnik G. (2016). Stroke and Heart Failure: Clinical Features, Access to Care, and Outcomes. J. Stroke Cerebrovasc. Dis..

[41] Bernhardt J., Raffelt A., Churilov L., Lindley R.I., Speare S., Ancliffe J., Katijjahbe M.A., Hameed S., Lennon S., McRae A. (2015). Exploring threats to generalisability in a large international rehabilitation trial (AVERT). BMJ Open.

[42] Stergiou N., Harbourne R., Cavanaugh J. (2006). Optimal movement variability: A new theoretical perspective for neurologic physical therapy. J. Neurol. Phys. Ther..

[43] Bruijn S.M., Meijer O.G., Beek P.J., van Dieën J.H. (2013). Assessing the stability of human locomotion: A review of current measures. J. R. Soc. Interface.

[44] Yentes J.M., Raffalt P.C. (2021). Entropy Analysis in Gait Research: Methodological Considerations and Recommendations. Ann. Biomed. Eng..

[45] Yentes J.M., Hunt N., Schmid K.K., Kaipust J.P., McGrath D., Stergiou N. (2013). The appropriate use of approximate entropy and sample entropy with short data sets. Ann. Biomed. Eng..

[46] Delgado-Bonal A., Marshak A. (2019). Approximate Entropy and Sample Entropy: A Comprehensive Tutorial. Entropy.

[47] Lubetzky A.V., Harel D., Lubetzky E. (2018). On the effects of signal processing on sample entropy for postural control. PLoS ONE.

[48] Harrison S.J., Stergiou N. (2015). Complex Adaptive Behavior and Dexterous Action. Nonlinear Dyn. Psychol. Life Sci..

[49] Richman J.S., Moorman J.R. (2000). Physiological time-series analysis using approximate entropy and sample entropy. Am. J. Physiol. Heart Circ. Physiol..

[50] Chen W., Zhuang J., Yu W., Wang Z. (2009). Measuring complexity using FuzzyEn, ApEn, and SampEn. Med. Eng. Phys..

[51] Chen W., Wang Z., Xie H., Yu W. (2007). Characterization of surface EMG signal based on fuzzy entropy. IEEE Trans. Neural Syst. Rehabil. Eng..

[52] Hornero R., Abásolo D., Escudero J., Gómez C. (2009). Nonlinear analysis of electroencephalogram and magnetoencephalogram recordings in patients with Alzheimer’s disease. Philos. Trans. A Math. Phys. Eng. Sci..

[53] Xie H.-B., Zheng Y.-P., Guo J.-Y., Chen X. (2010). Cross-fuzzy entropy: A new method to test pattern synchrony of bivariate time series. Inf. Sci..

[54] Jin S.H., Lin P., Hallett M. (2010). Linear and nonlinear information flow based on time-delayed mutual information method and its application to corticomuscular interaction. Clin. Neurophysiol..

[55] Ahmed M.U., Mandic D.P. (2011). Multivariate multiscale entropy: A tool for complexity analysis of multichannel data. Phys. Rev. E.

[56] Busa M.A., van Emmerik R.E.A. (2016). Multiscale entropy: A tool for understanding the complexity of postural control. J. Sport. Health Sci..

[57] Costa M., Goldberger A.L., Peng C.K. (2005). Multiscale entropy analysis of biological signals. Phys. Rev. E.

[58] Kantelhardt J.W., Zschiegner S.A., Koscielny-Bunde E., Havlin S., Bunde A., Stanley H.E. (2002). Multifractal detrended fluctuation analysis of nonstationary time series. Phys. A Stat. Mech. Appl..

[59] Marmelat V., Torre K., Beek P.J., Daffertshofer A. (2014). Persistent fluctuations in stride intervals under fractal auditory stimulation. PLoS ONE.

[60] Rhea C.K., Kiefer A.W., Wittstein M.W., Leonard K.B., MacPherson R.P., Wright W.G., Haran F.J. (2014). Fractal gait patterns are retained after entrainment to a fractal stimulus. PLoS ONE.

[61] Phinyomark A., Larracy R., Scheme E. (2020). Fractal Analysis of Human Gait Variability via Stride Interval Time Series. Front. Physiol..

[62] Liu K., Wang H., Xiao J., Taha Z. (2015). Analysis of human standing balance by largest lyapunov exponent. Comput. Intell. Neurosci..

[63] Mehdizadeh S. (2018). The largest Lyapunov exponent of gait in young and elderly individuals: A systematic review. Gait Posture.

[64] Toebes M.J., Hoozemans M.J., Furrer R., Dekker J., van Dieën J.H. (2012). Local dynamic stability and variability of gait are associated with fall history in elderly subjects. Gait Posture.

[65] Mehdizadeh S., Sanjari M.A. (2017). Effect of noise and filtering on largest Lyapunov exponent of time series associated with human walking. J. Biomech..

[66] Cignetti F., Decker L.M., Stergiou N. (2012). Sensitivity of the Wolf’s and Rosenstein’s algorithms to evaluate local dynamic stability from small gait data sets. Ann. Biomed. Eng..

[67] Moraiti C.O., Stergiou N., Vasiliadis H.S., Motsis E., Georgoulis A. (2010). Anterior cruciate ligament reconstruction results in alterations in gait variability. Gait Posture.

[68] Ahn J., Hogan N. Is estimation of Floquet multipliers of human walking valid?. Proceedings of the 2014 40th Annual Northeast Bioengineering Conference (NEBEC).

[69] Caballero C., Barbado D., Moreno F. (2014). Non-linear tools and methodological concerns measuring human movement variability: An overview. Eur. J. Hum. Mov..

[70] Müller W., Jung A., Ahammer H. (2017). Advantages and problems of nonlinear methods applied to analyze physiological time signals: Human balance control as an example. Sci. Rep..

[71] Wijnants M.L., Bosman A.M., Hasselman F., Cox R.F., Van Orden G.C. (2009). 1/f scaling in movement time changes with practice in precision aiming. Nonlinear Dyn. Psychol. Life Sci..

[72] Costa M., Priplata A.A., Lipsitz L.A., Wu Z., Huang N.E., Goldberger A.L., Peng C.K. (2007). Noise and poise: Enhancement of postural complexity in the elderly with a stochastic-resonance-based therapy. Europhys. Lett..

[73] Kwakkel G., Stinear C., Essers B., Munoz-Novoa M., Branscheidt M., Cabanas-Valdés R., Lakičević S., Lampropoulou S., Luft A.R., Marque P. (2023). Motor rehabilitation after stroke: European Stroke Organisation (ESO) consensus-based definition and guiding framework. Eur. Stroke J..

[74] Ozturk A., Tartar A., Ersoz Huseyinsinoglu B., Ertas A.H. (2016). A clinically feasible kinematic assessment method of upper extremity motor function impairment after stroke. Measurement.

[75] Hedman L.D., Quinn L., Gill-Body K., Brown D.A., Quiben M., Riley N., Scheets P.L. (2018). White Paper: Movement System Diagnoses in Neurologic Physical Therapy. J. Neurol. Phys. Ther..

[76] Quinn L., Riley N., Tyrell C.M., Judd D.L., Gill-Body K.M., Hedman L.D., Packel A., Brown D.A., Nabar N., Scheets P. (2021). A Framework for Movement Analysis of Tasks: Recommendations from the Academy of Neurologic Physical Therapy’s Movement System Task Force. Phys. Ther..

[77] Kyvelidou A., Kurz M.J., Ehlers J.L., Stergiou N. (2008). Aging and partial body weight support affects gait variability. J. Neuroeng. Rehabil..

[78] Hollman J.H., Watkins M.K., Imhoff A.C., Braun C.E., Akervik K.A., Ness D.K. (2016). Complexity, fractal dynamics and determinism in treadmill ambulation: Implications for clinical biomechanists. Clin. Biomech..

[79] Terrier P., Dériaz O. (2011). Kinematic variability, fractal dynamics and local dynamic stability of treadmill walking. J. Neuroeng. Rehabil..

[80] Sloot L.H., van Schooten K.S., Bruijn S.M., Kingma H., Pijnappels M., van Dieën J.H. (2011). Sensitivity of local dynamic stability of over-ground walking to balance impairment due to galvanic vestibular stimulation. Ann. Biomed. Eng..

[81] Paci M., Nannetti L., Casavola D., Lombardi B. (2016). Differences in motor recovery between upper and lower limbs: Does stroke subtype make the difference?. Int. J. Rehabil. Res..

[82] Hamzat T.K., Owolabi M.O., Vincent-Onabajo G.O. (2014). Trajectory of Motor Performance over Twelve Months in Nigerian Stroke Survivors. Brain Impair..

[83] Skurvydas A., Juodzbaliene V., Darbutas T., Brazaitis M. (2018). One year after ischemic stroke: Changes in leg movement path stability in a speed-accuracy task but no major effects on the hands. Hum. Mov. Sci..

[84] Kong K.H., Lee J. (2013). Temporal recovery and predictors of upper limb dexterity in the first year of stroke: A prospective study of patients admitted to a rehabilitation centre. NeuroRehabilitation.

[85] Rand D., Eng J.J. (2012). Disparity between functional recovery and daily use of the upper and lower extremities during subacute stroke rehabilitation. Neurorehabil. Neural Repair..

[86] Broeks J., Lankhorst G., Rumping K., Prevo A. (1999). The long-term outcome of arm function after stroke: Results of a follow-up study. Disabil. Rehabil..

[87] Ryu H.X., Kuo A.D. (2021). An optimality principle for locomotor central pattern generators. Sci. Rep..

[88] Minassian K., Hofstoetter U.S., Dzeladini F., Guertin P.A., Ijspeert A. (2017). The Human Central Pattern Generator for Locomotion: Does It Exist and Contribute to Walking?. Neuroscientist.

[89] Klarner T., Zehr E.P. (2018). Sherlock Holmes and the curious case of the human locomotor central pattern generator. J. Neurophysiol..

[90] Kathe C., Skinnider M.A., Hutson T.H., Regazzi N., Gautier M., Demesmaeker R., Komi S., Ceto S., James N.D., Cho N. (2022). The neurons that restore walking after paralysis. Nature.

[91] Corazza S., Mündermann L., Gambaretto E., Ferrigno G., Andriacchi T. (2010). Markerless Motion Capture through Visual Hull, Articulated ICP and Subject Specific Model Generation. Int. J. Comput. Vis..

[92] Hamacher D., Singh N.B., Van Dieën J.H., Heller M.O., Taylor W.R. (2011). Kinematic measures for assessing gait stability in elderly individuals: A systematic review. J. R. Soc. Interface.

[93] Usmani S., Saboor A., Haris M., Khan M.A., Park H. (2021). Latest Research Trends in Fall Detection and Prevention Using Machine Learning: A Systematic Review. Sensors.

[94] Drapeaux A., Carlson K. (2020). A comparison of inertial motion capture systems: DorsaVi and Xsens. Int. J. Kinesiol. Sports Sci..

[95] Chen S., Lach J., Lo B., Yang G.Z. (2016). Toward Pervasive Gait Analysis with Wearable Sensors: A Systematic Review. IEEE J. Biomed. Health Inform..

[96] Maletsky L.P., Sun J., Morton N.A. (2007). Accuracy of an optical active-marker system to track the relative motion of rigid bodies. J. Biomech..

[97] van der Kruk E., Reijne M.M. (2018). Accuracy of human motion capture systems for sport applications; state-of-the-art review. Eur. J. Sport. Sci..

[98] Stancic I., Supuk T., Panjkota A. (2013). Design, development and evaluation of optical motion-tracking system based on active white light markers. IET Sci. Meas..

[99] Della Croce U., Leardini A., Chiari L., Cappozzo A. (2005). Human movement analysis using stereophotogrammetry. Part 4: Assessment of anatomical landmark misplacement and its effects on joint kinematics. Gait Posture.

[100] Kainz H., Modenese L., Lloyd D.G., Maine S., Walsh H.P.J., Carty C.P. (2016). Joint kinematic calculation based on clinical direct kinematic versus inverse kinematic gait models. J. Biomech..

[101] Mantovani G., Lamontagne M. (2017). How Different Marker Sets Affect Joint Angles in Inverse Kinematics Framework. J. Biomech. Eng..

[102] Preatoni E., Bergamini E., Fantozzi S., Giraud L.I., Orejel Bustos A.S., Vannozzi G., Camomilla V. (2022). The Use of Wearable Sensors for Preventing, Assessing, and Informing Recovery from Sport-Related Musculoskeletal Injuries: A Systematic Scoping Review. Sensors.

[103] Colyer S.L., Evans M., Cosker D.P., Salo A.I.T. (2018). A Review of the Evolution of Vision-Based Motion Analysis and the Integration of Advanced Computer Vision Methods towards Developing a Markerless System. Sports Med. Open.

[104] Collins K.C., Kennedy N.C., Clark A., Pomeroy V.M. (2018). Kinematic Components of the Reach-to-Target Movement after Stroke for Focused Rehabilitation Interventions: Systematic Review and Meta-Analysis. Front. Neurol..

[105] Roggio F., Ravalli S., Maugeri G., Bianco A., Palma A., Di Rosa M., Musumeci G. (2021). Technological advancements in the analysis of human motion and posture management through digital devices. World J. Orthop..

[106] Rabelo M., Nunes G.S., da Costa Amante N.M., de Noronha M., Fachin-Martins E. (2016). Reliability of muscle strength assessment in chronic post-stroke hemiparesis: A systematic review and meta-analysis. Top. Stroke Rehabil..

[107] Ekstrand E., Lexell J., Brogårdh C. (2015). Isometric and isokinetic muscle strength in the upper extremity can be reliably measured in persons with chronic stroke. J. Rehabil. Med..

[108] Kwong P.W.H., Ng S.S.M., Chung R.C.K., Ng G.Y.F. (2017). A structural equation model of the relationship between muscle strength, balance performance, walking endurance and community integration in stroke survivors. PLoS ONE.

[109] Rabelo M., Fachin-Martins E. (2018). Inter-rater and test/retest reliabilities of the isokinetic measurements: Assessing strength and endurance of the trunk muscles in two different protocols for able-bodied and post-stroke hemiparesis. Top. Stroke Rehabil..

[110] Abdollahi I., Taghizadeh A., Shakeri H., Eivazi M., Jaberzadeh S. (2015). The relationship between isokinetic muscle strength and spasticity in the lower limbs of stroke patients. J. Bodyw. Mov. Ther..

[111] Karthikbabu S., Chakrapani M. (2017). Hand-Held Dynamometer is a Reliable Tool to Measure Trunk Muscle Strength in Chronic Stroke. J. Clin. Diagn. Res..

[112] Merletti R., Temporiti F., Gatti R., Gupta S., Sandrini G., Serrao M. (2023). Translation of surface electromyography to clinical and motor rehabilitation applications: The need for new clinical figures. Transl. Neurosci..

[113] Belfatto A., Scano A., Chiavenna A., Mastropietro A., Mrakic-Sposta S., Pittaccio S., Molinari Tosatti L., Molteni F., Rizzo G. (2018). A Multiparameter Approach to Evaluate Post-Stroke Patients: An Application on Robotic Rehabilitation. Appl. Sci..

[114] Fang C., He B., Wang Y., Cao J., Gao S. (2020). EMG-Centered Multisensory Based Technologies for Pattern Recognition in Rehabilitation: State of the Art and Challenges. Biosensors.

[115] Pierella C., Pirondini E., Kinany N., Coscia M., Giang C., Miehlbradt J., Magnin C., Nicolo P., Dalise S., Sgherri G. (2020). A multimodal approach to capture post-stroke temporal dynamics of recovery. J. Neural Eng..

[116] Hallett M., DelRosso L.M., Elble R., Ferri R., Horak F.B., Lehericy S., Mancini M., Matsuhashi M., Matsumoto R., Muthuraman M. (2021). Evaluation of movement and brain activity. Clin. Neurophysiol..

[117] Maura R.M., Rueda Parra S., Stevens R.E., Weeks D.L., Wolbrecht E.T., Perry J.C. (2023). Literature review of stroke assessment for upper-extremity physical function via EEG, EMG, kinematic, and kinetic measurements and their reliability. J. Neuroeng. Rehabil..

