# Supplementary material for: Biomechanical Assessment Methods Used in Chronic Stroke: A Scoping Review of Non-Linear Approaches
Source: Sensors (Basel). 2024 Apr 6;24(7):2338. doi: 10.3390/s24072338 (PMC11014015; doi:10.3390/s24072338)
Supplement: Supplementary file 1 [file sensors-24-02338-s001.zip › sensors-2898355-supplementary.pdf]

Biomechanical Assessment Methods Used in Chronic Stroke: A Protocol for a Scoping Review of Non-linear Approach

https://doi.org/10.17605/OSF.IO/R3PE9

Prepared for Registration to Open Science Framework

Submitted 28/07/2023

Departamento de Tecnologias de Diagnóstico e Terapêutica
CESPU: Escola Superior de Saúde do Vale do Ave
Vila Nova de Famalicão, Portugal

Marta Freitas
marta.goncalves@ipsn.cespu.pt

REVIEW TITLE

Biomechanical Research Methods Used in Chronic Stroke: Protocol for a Scoping Review of a Non-linear Approach

TYPE OF REVIEW

Scoping Review.

STAGE OF REVIEW AT TIME OF THIS SUBMISSION

Ongoing: Piloting of the study selection process.

ORGANISATIONAL AFFILIATION OF THE REVIEW

CESPU: Escola Superior de Saúde do Vale do Ave

University of Porto

REVIEW TEAM MEMBERS AND AFFILIATION

Marta Freitas

Escola Superior de Saúde do Vale do Ave (ESSVA), Cooperativa de Ensino Superior Politécnico e Universitário (CESPU), Vila Nova de Famalicão, Portugal; H²M - Health and Human Movement Unit, Instituto Politécnico de Saúde do Norte, CESPU, Vila Nova de Famalicão, Portugal; Research Centre in Physical Activity, Health and Leisure, Faculty of Sport, University of Porto, Porto, Portugal

Electronic address: marta.goncalves@ipsn.cespu.pt

Augusta Silva

Escola Superior de Saúde (ESS), Instituto Politécnico do Porto, Área Científica de Fisioterapia, Centro de Investigação em Reabilitação - Centro de Estudos de Movimento e Atividade Humana, Porto, PORTUGAL

Electronic address: augusta.silva@ess.ipp.pt

João Paulo Vilas-Boas

Centre for Research, Education, Innovation and Intervention in Sport (CIFI2D), Faculty of Sports, University of Porto, 4200-250 Porto, Portugal, Porto Biomechanics Laboratory (LABIOMEP), 4200-450 Porto, Portugal
Electronic address: jpvb@fade.up.pt

Francisco Pinho
Escola Superior de Saúde do Vale do Ave (ESSVA), Cooperativa de Ensino Superior Politécnico e Universitário (CESPU), Vila Nova de Famalicão, Portugal; H²M - Health and Human Movement Unit, Instituto Politécnico de Saúde do Norte, CESPU, Vila Nova de Famalicão, Portugal
Electronic address: francisco.pinho@ipsn.cespu.pt

Liliana Pinho
Escola Superior de Saúde do Vale do Ave (ESSVA), Cooperativa de Ensino Superior Politécnico e Universitário (CESPU), Vila Nova de Famalicão, Portugal; H²M -Health and Human Movement Unit, Instituto Politécnico de Saúde do Norte, CESPU, Vila Nova de Famalicão, Portugal; Research Centre in Physical Activity, Health and Leisure, Faculty of Sport, University of Porto, Porto, Portugal.
Electronic address: liliana.pinho@ipsn.cespu.pt

Sandra Silva
Escola Superior de Saúde do Vale do Ave (ESSVA), Cooperativa de Ensino Superior Politécnico e Universitário (CESPU), Vila Nova de Famalicão, H²M - Health and Human Movement Unit, Instituto Politécnico de Saúde do Norte, CESPU, Portugal; School of Health Sciences (ESSUA) and Department of Medical Sciences (DCM), University of Aveiro, Aveiro, Portugal.
Electronic address: sandra.silva@ipsn.cespu.pt

Vânia Figueira
Escola Superior de Saúde do Valedo Ave (ESSVA), Cooperativa de Ensino Superior Politécnico e Universitário (CESPU), Vila Nova de Famalicão, H²M -Health and Human Movement Unit,

Instituto Politécnicode Saúde do Norte, CESPU; Famalicão,Portugal; Research Centre in Physical Activity, Health and Leisure, Faculty of Sport, University of Porto, Porto, Portugal. Electronic address: vania.figueira@ipsn.cespu.pt

REVIEW METHODS

This scoping review will be conducted in accordance with the Preferred Reporting Items for Systematic Reviewers and Meta-Analysis extension for Scoping Reviews (PRISMA-ScR) framework (Tricco et al, 2018).

Review questions

The main review question is "What are the nonlinear measures used in processing kinematic and kinetic data in the assessment of human movement after chronic stroke?"

The review sub-questions are listed as follows:

1. What laboratorial instruments were used to collect kinematic and kinetic data in the identified studies?
2. What kinematic and kinetic variables were considered in the identified studies?
3. Which tasks were covered in the identified studies?

Eligibility Criteria

Eligibility criteria were established a priori using the acronym PCC (Population, Concept, Context), in accordance with Joanna Briggs Institute methodology (Peters et al., 2020).

Population: Chronic Poststroke adults (>19 years old) (≥19 years old)

Concept: Non-linear measures in kinetic or kinematic data processing of human movement analysis

Context: Open

Studies are also eligible if they meet the following criteria:

- Experimental and epidemiological study designs.
- Studies published in Portuguese and English.

Studies will be excluded if they have any of the following characteristics:

- Systematic, narrative or scoping reviews will not be included to avoid duplication of data.
- Letters, editorials and qualitative method designs.

Information Source

The relevant studies will be identified by searching the databases PubMed®, Web of Science®, Institute of Electrical and Electronics Engineers®(IEEE) and Science Direct®, from 2013 until April 2023. The Google Scholar will be contemplated as unpublished and gray literature. The reference lists of original research articles and reviews on the topic will be manually checked to identify other eligible studies. The search strategy for different databases is presented in Table S1, according to their specificities. Two reviewers, independently, will carry out the search.

Table S1: Initial search for different databases

Database	Entry Terms
Pubmed®	Stroke AND (measure OR measurement OR evaluation OR analysis OR assessment) AND (non-linear OR nonlinear OR entropy OR Lyapunov OR "nonlinear variables" OR tools OR dynamic OR variability) AND ("human movement" OR motion) NOT (EEG OR Cardiac).
Institute of Electrical and Electronics Engineers®	Stroke AND (nonlinear OR entropy OR Lyapunov OR variability) NOT EEG
Web of Science®	Stroke AND (non-linear OR entropy OR Lyapunov) AND ("human movement")
Science Direct®	Stroke AND (non-linear OR entropy OR Lyapunov) AND ("human movement") NOT (EEG OR Cardiac)
Google Scholar®	Stroke AND "non-linear measure"

Based on eligibility criteria defined a priori, an analysis of titles/abstracts will be carried out independently by the 2 reviewers. After discussion of the results of this calibration exercise and consensus has been reached, changes will be made to the eligibility criteria if necessary. The researchers will start the screening process only when there is a consensus of at least 75%.

After the search, all identified records will be imported to Endnote software (Clarivate) and duplicates will be removed. The titles and abstracts will be screened by two reviewers, after which they will categorize studies as "include" or "exclude". This stage will allow identifying articles for full-text screening. The reasons why the studies will be excluded will be detailed in the final scoping review.

The results of the search will be presented in a PRISMA-ScR flowchart.

Data Extraction

Data will be extracted for the authors, year of publication, study design, characteristics of the participants (n, gender, age, side lesion, stroke type, time post-stroke), the tasks under study, assessment instruments, kinetic and kinematic variables, and nonlinear measures. If necessary, the corresponding authors of published articles will be contacted by e-mail to request missing or additional data. Two authors will independently extract the abovementioned data by using a draft charting table adapted from the original JBI template. The data extraction will be performed independently by the 2 reviewers, while disagreements will be resolved with a third author.

Critical appraisal of individual sources of evidence

We will not conduct quality appraisal, since this is a scoping review, which is in accordance with Preferred Reporting Items for Systematic reviews and Meta-Analyses extension for Scoping Reviews and Joanna Briggs Institute methodology.

Data Presentation

The full scoping review will be reported in accordance with the PRISMA-ScR checklist. A narrative report will be produced to summarize the extracted data around the following outcomes: tasks, assessment instruments, kinetic and kinematic variables, and nonlinear measures. These results will be described in relation to the research question and in the context of the overall study purpose. Gap identification will detect areas, such as tasks and contexts that lack data on the nonlinear measures to report kinematic and kinetic data, and if there is limited data among post stroke individuals. To summarize the evidence on specific topics, all the results will be presented in 2 tables, by two main conceptual categories, the

kinetic and kinematic data and non-linear measures. A synthesis of the main findings will accompany the tabulated data.

KEYWORDS

CHRONIC STROKE, ASSESSMENT, NON-LINEAR, KINETIC, KINEMATIC

AUTHOR´S CONTRIBUTIONS

Authors MF and LP conceived the idea of the scoping review and developed the research questions.

FP and JPVB provided conceptual expertise of the kinematic and kinetic assessment. MF, LP, AS and SS contributed meaningfully to drafting and editing the manuscript and developing the study methods. MF and LP contributed to the search strategies and created the search strategy table.

FUNDING SOURCES

None.

CONFLICT OF INTEREST

None Declared.

COUNTRY

Portugal
